# The mortality of companies

**DOI:** 10.1098/rsif.2015.0120

**Published:** 2015-05-06

**Authors:** Madeleine I. G. Daepp, Marcus J. Hamilton, Geoffrey B. West, Luís M. A. Bettencourt

**Affiliations:** 1 Santa Fe Institute, Santa Fe, NM, USA; 2 Integrated Studies in Land and Food Systems, University of British Columbia, Vancouver, British Columbia, Canada; 3 School of Human Evolution and Social Change, Arizona State University, Tempe, AZ, USA; 4 Department of Mathematics, Imperial College London, London, UK

**Keywords:** firm longevity, mergers and acquisitions, survival analysis, stock markets

## Abstract

The firm is a fundamental economic unit of contemporary human societies. Studies on the general quantitative and statistical character of firms have produced mixed results regarding their lifespans and mortality. We examine a comprehensive database of more than 25 000 publicly traded North American companies, from 1950 to 2009, to derive the statistics of firm lifespans. Based on detailed survival analysis, we show that the mortality of publicly traded companies manifests an approximately constant hazard rate over long periods of observation. This regularity indicates that mortality rates are independent of a company's age. We show that the typical half-life of a publicly traded company is about a decade, regardless of business sector. Our results shed new light on the dynamics of births and deaths of publicly traded companies and identify some of the necessary ingredients of a general theory of firms.

## 1. Introduction

Publicly traded companies are among the most important economic units of contemporary human societies [1–6]. As of 2011, the total market capitalization of firms in the New York Stock Exchange was 14.24 trillion dollars, comparable to the entire gross domestic product of the USA. While researchers have devoted considerable attention to the distribution of firm size [7–11], the distribution of firm lifespan has been the subject of far fewer studies [12]. Thus, despite the availability of much quantitative information, our understanding of the way public companies live and die remains limited.

At present, there are several arguments addressing the statistics of company lifespans that have led researchers to a range of different conclusions. Some of these considerations hinge on the interpretation of the meaning of the death event for a company. In the framework of this paper, definitions of ‘birth’ and ‘death’ are based on the sales reports available in the *Compustat* database; details can be found in §4. While liquidation is often responsible for firm deaths, a much more common cause of death relates to the disappearance of companies through mergers and acquisitions. Thus, in our definition, firms may ‘die’ through a variety of processes: they may split, merge or liquidate as economic and technological conditions change. This raises the question of what characteristics of firms may initiate such events. In particular, it has often been suggested that the mortality rates of firms are age-dependent [5,13–16], a proposition that offers significant insight into the forces that determine firm survival. We address this question using a comprehensive database of over 25 000 publicly traded North American companies covering a large spectrum of business sectors over the period 1950–2009. The present analysis provides one of the largest studies of this kind [5,6], both in terms of numbers of firms and timespan.

There is a great diversity of perspectives on a theory of the firm, focusing on different aspects of their costs, organization and evolution. In modern economic theory, the existence and boundaries of firms are understood in counterpoint to the dynamics of self-organization in markets. Economists such as Coase [3,17,18] and Williamson [19,20] proposed that firms exist in order to minimize (positive) market *transaction costs* involved in the production of goods and services. In situations when, for example, there is particular specificity of goods and services exchanged between two economic agents, such transactions may be best organized internally to an organization rather than negotiated in the open market [2,20–24]. As such, firms may split, merge or liquidate in response to economic agents evolving new and better ways of dealing with the various costs and revenues of production and exchange [21–24]. Therefore, at least on the average, the merger of existing companies should be approximately neutral in terms of the balance between costs and benefits [21–24]. However, this relatively simple picture becomes more complex in the light of behavioural studies of the impact of decision-making and management practices on the growth and viability of actual firms [25,26].

A perspective more directly tied to the demography of companies is organizational ecology [4,5]. In the framework of organizational ecology, organizations that vary in their structure and relationships are modelled as competing for finite resources within a complex ecology of economic interactions [27,28]. In this approach, which emerged from economic sociology, companies are seen as units of selection in markets and their longevity is the result of their successes of learning and adaptation in these environments. Similar to this approach, we employ mathematical models from theoretical ecology to examine the lifespans and mortality of companies.

Among the most widely replicated results relating to the mortality of firms is Stinchcombe's [13] *liability of newness.* This is the expectation that young establishments experience higher mortality rates. This scenario is supported by observation of US manufacturing plants, Argentinian and Irish newspaper companies and other types of businesses [14,15]. Theoretical grounding draws from the adaptive requirements of market entry; it takes time for young companies to gain the competencies and build relationships that will ensure their ability to survive [29,30]. Moreover, new companies are likely to be smaller and less experienced and thus more susceptible to market shocks [29]. Knott & Posen [31] stress the evolutionary character of these arguments by suggesting that liability of newness is evidence for market-based selection.

However, more recent evidence begins to diverge from this hypothesis. In a study of West German business enterprises, Bruderl & Schussler [16] find that companies are, in fact, protected from mortality in the immediate period after founding. This *liability of adolescence* likely results from the buffer a firm acquires via its capital endowment at birth [29], which is also a characteristic of firms that have recently entered financial markets. As their initial capital stock is expended, less profitable companies become more vulnerable to environmental changes in market conditions.

A third perspective suggests that mortality rates increase as companies age. This idea is based upon two related concepts: the first is *liability of senescence*, the idea that as companies age, they accumulate rules and stagnating relationships with consumers and input markets that render them less agile and that re-configuration is increasingly expensive [32]. Arguing instead for a *liability of obsolescence*, Sorenson & Stuart [33] suggest that environmental requirements change over time and that, although firms may improve in competence and efficiency with age by becoming more specialized, these specific adaptations also increase the companies’ risk to new kinds of external shocks that will inevitably beset them.

Finally, Coad [12] has argued that these assorted liabilities constitute small deviations, at the tails, from an aggregate lifespan distribution that is generally well approximated by an exponential distribution. This proposition has been confirmed in Italian, Spanish and French firms [34]. As noted by Amaral *et al.* [9] and Coad [35], the statistical patterns of firm entry and exit will affect the distribution of firm sizes in any given year and set its form and temporal stability. Thus, a better understanding of the mortality risk of firms is necessary to generate new insights on the empirically observed scaling regularities in firm size frequency distributions [8,11].

In this paper, we test these alternative hypotheses of firm lifespan and mortality risk by analysing a large database [36] of North American publicly traded companies between 1950 and 2009. We confirm the hypothesis of an approximately constant mortality rate, finding that the exponential distribution of firm lifespans holds across business sectors and causes of mortality. We apply survival analysis to estimate in a variety of ways that the firms in our dataset have a half-life of approximately 10 years, regardless of age.

## 2. Results

**Figure 1.**
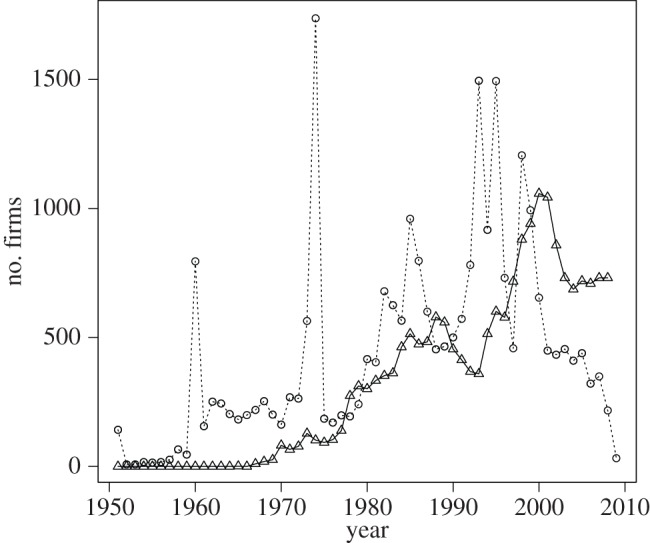
Number of firm births and deaths in each year. We observe that the number of firms entering (births, circles) and exiting (deaths, triangles) North American stock markets varies significantly over time, reflecting in part economic cycles. Note that before 1975 very few firms die, reflecting a survival bias in the *Compustat* dataset. Similarly, there are two spikes in births in 1960 and in 1974 that may be reflective of changes in the *Compustat* database or the conditions of market entry, not in the patterns we seek to analyse. We limited much of our analysis to the period after 1975 to control for this bias.

Details of our datasets and definition of firm ‘births’, ‘deaths’, ‘lifespans’ and ‘half-lives’ are provided in Material and methods. Annual numbers of entries (births) and exits (deaths) for publicly traded firms in the *Compustat* database [36] are shown in figure 1. The number of births and deaths per year in this dataset varies substantially over time, reflecting, in part, general economic conditions. We note that the number of deaths before 1975 is very low, suggesting potential survival bias over this period towards longer lifetimes. We address this issue in detail below by analysing the full dataset as well as one constrained by selectively excluding the period before 1975.

**Figure 2.**
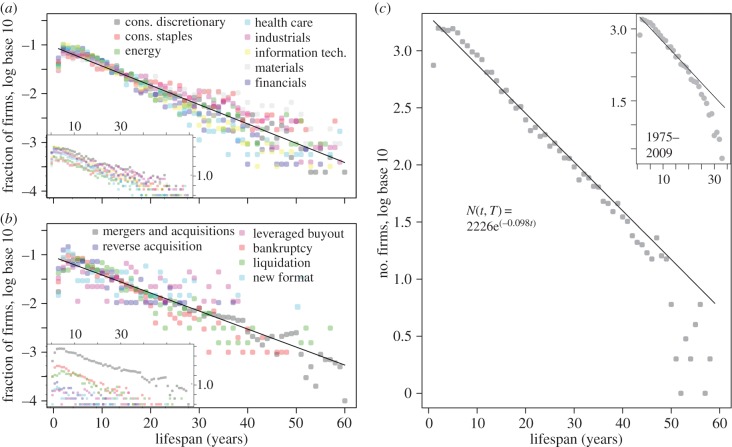
Frequency distribution of firm lifespans. The frequency distribution of firm lifespans is approximately exponential, independent of business sector. Colours denote firms from different economic sectors (*a*) and with different reasons of death (*b*) for the period 1950–2009. Insets show the lifespan frequency distributions before normalization by sector size. In (*b*), the reasons ‘other’ and privatization were omitted; in (*a*), the telecommunications, utilities and transportation sectors were omitted based on small sample size. The aggregate distributions are fit by a simple exponential function shown in (*c*). For the full window, the fit is *N*(*t*, *T*) = 2226e^−*λt*^ with *λ* = 0.098 and 95% confidence interval 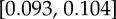. For the constrained window, the fit is *N*(*t*, *T*) = 2279e^−*λt*^ with *λ* = 0.131 and 95% confidence interval 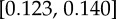.

We give a first impression of the statistics of company lifespans in figure 2. Figure 2*a* shows the frequency distribution of lifespans for all firms that were born and died within the period 1950–2009 by broad economic sector. Figure 2*b* shows a similar frequency plot where colours denote the most common causes of mortality (table 1). Figure 2*c* shows that an exponential distribution of lifespans is a reasonable fit to data (solid lines), either restricted to the period of 1975 onwards or not.

**Table 1.** Leading causes of death in publicly traded companies 1950–2009.

cause of death	mortality (%)
mergers and acquisitions	45.1
other	28.0
unlisted	15.2
bankruptcy	4.5
liquidation	3.5
privatization	2.8
reverse acquisition	0.4
leveraged buyout	0.4
new format	0.4

We also observe in all panels of figure 2 that fewer firms die in the first few years of entering the market than a purely exponential distribution would suggest. This provides initial evidence for the liability of adolescence. In the following, we perform a set of more rigorous statistical analyses to test the idea of a constant death rate as a function of age and to provide a set of estimates of the half-lives of publicly traded firms.

### 2.1. Constant death rate and exponentially distributed lifespans

The simplest conceptual framework for understanding the distribution of lifespans is inspired by a decay process in which the decay rate is assumed to be proportional to the number of remaining constituents. For firms, this translates into the assumption that the number of deaths, Δ*N_d_*(*t*, *T*), occurring in some small discrete time interval from *t* to *t* + Δ*t* is proportional to the number of companies remaining alive at time *t*, *N*(*t*, *T*), out of an initial cohort of *N*(0, *T*) firms at time *t* = 0. *T* denotes the time window of observation, which can be arbitrarily varied within the total timespan covered by the dataset. In the present case *T* ≤ 60 years. Thus,2.1
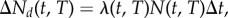
where *λ* is the exit (or hazard) rate, which in general depends on both *t* and *T*. Since the number of firms remaining alive at time *t* is *N*(*t*, *T*) = *N*(0, *T*) − *N_d_*(*t*, *T*), Δ*N_d_*(*t*, *T*) =−Δ*N*(*t*, *T*) if the time window, *T*, is kept fixed. In the limit of continuous time (Δ*t* → 0), this leads to2.2
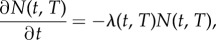
whose general solution is given by2.3
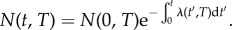
If *λ* is independent of *t*, but not necessarily of *T*, this reduces to the classic exponential form *N*(*t*, *T*) = *N*(0, *T*)e*^−*λ*^*^(*T*)*t*^ which, as discussed above, is a good fit to the data.

By selecting firms whose lifespans are less than *T* such that the entire cohort lives and dies within the time window, *T*, we can interpret *N*(*t*, *T*) as the frequency distribution of companies with lifespans *t* ≤ *T*. However, in our dataset, time is discrete (data are reported on an annual basis) leading to a subtlety on how to enforce boundary conditions at *t* = 0 and *t* = *T*. By construction, all firms in the cohort are dead at *t* = *T*, so *N*(*T*, *T*) = 0. Equation (2.1) would then require Δ*N_d_*(*T*, *T*) = 0, which would prohibit further mortality when the window is extended from *T* to *T* + 1 years. The difficulty reflects the observation that the solution to equation (2.2) cannot accommodate the boundary condition *N*(*T*, *T*) = 0 for *any* finite value of *λ*(*t*, *T*). We therefore need to modify the equation to ensure that ∂*N*(*T*, *T*)/∂*t* ≠ 0.

**Figure 3.**
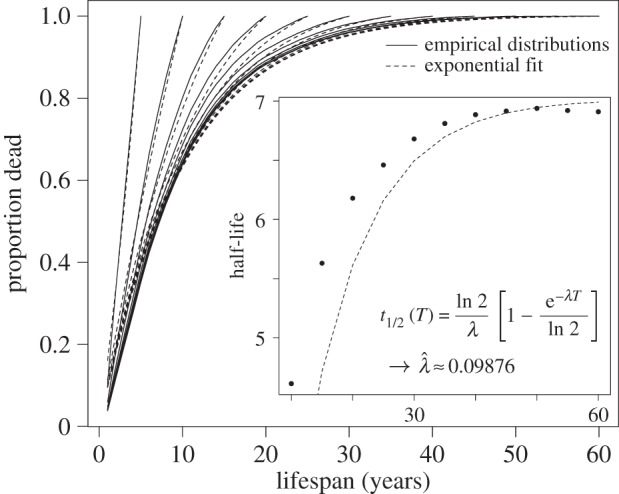
Exponential fit to firm mortality. An exponential mortality curve (dashed line), with appropriate boundary conditions, is fit to sets of firms that are born and die within an observation window, *T* (solid line). The exponential curve is a good fit across observation windows from a few years to several decades. The half-life estimated from each curve increases with *T* as shown in the inset. Its limiting value for large observation windows, *T* → *∞*, is approximately 7.02 years.

The simplest way of enforcing the correct boundary condition is to modify equation (2.1) to read2.4
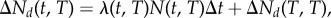
in which case equation (2.2) becomes2.5
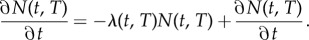
When *λ* is independent of *t* (though still a function of *T*) this can be solved to give2.6
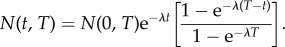
When 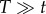 this reduces to the usual exponential formula: *N*(*t*, *T*) = *N*(0, *T*)e*^−*λ*t^*. The corresponding cumulative distribution function, *M*(*t*), for the fraction of companies that have died by time *t* within the observation window *T* is given by2.7
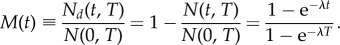
Fits to data at successively larger *T* are shown in figure 3. We observe that the modified exponential, equation (2.7), provides very good agreement with data over a broad range of values of *T*. From these fits, we can compute the *half-life* of firms, *t*_1/2_, defined as the time taken for half of the original cohort, *N*(0, *T*), to die (see electronic supplementary material, figure S2). This is determined by solving *M*(*t*_1/2_) = 1/2, which results in2.8
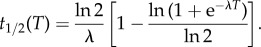
For 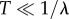 this gives 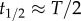, whereas for
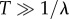
2.9
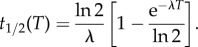
From half-life estimates across varying time windows equation (2.8) leads, for large *T*, to a hazard rate *λ* ≈ 0.099 yr^−1^, corresponding to an asymptotic half-life of about 7 years (see figure 3 (inset) and electronic supplementary material, figure S1).

This analysis provides a starting point for estimating the lifespan of firms. However, the assumption that the hazard rate, *λ*, is independent of *t* for each time window *T* necessarily implies that all firms have finite lifespans and therefore presumes *a priori* that they all eventually die (see Discussion). Furthermore, our half-life estimate appears low; if we look at all firms born in a particular year, it often takes longer than 7 years for half of them to disappear. In 1975, for example, it took almost 12 years for half of the cohort to die. When we omit those firms that do not die before 2009, we reach the half-life more than 2 years earlier.

This is very likely due to the omission of firms whose lifespans exceed the entire window of observation, leading to a bias towards early mortality in our half-life estimate. A more complete analysis therefore needs to include *censored* firms, i.e. those that were already alive at the beginning of the observation window and/or that remained alive at the end. For these companies, we know that their lifespan is at least as long as, and likely longer than the period over which they appear in the dataset (right-censored). This involves a large fraction of the companies in the *Compustat* dataset: in the 60 years covered, 6873 firms were still alive at the end of the observation window, compared with nearly a third still alive at the end of the constrained 40-year window. To address the issue of how to compute firm lifespans that include these censored companies, we now turn to methods of statistical survival analysis [37].

### 2.2 Survival analysis

Survival analysis is a well-developed field of applied statistics [37] that starts with the definition of a survival function, *S*(*t*). The survival function is the cumulative probability of a firm being alive after time *t*. For a cohort of firms born at an initial time, *t* = 0, *S*(*t*) is expressed as the fraction of firms still alive at time *t*: *S*(*t*) = *N*(*t*)/*N*(0). (For ease of presentation, we suppress any explicit dependence on the time window of observation.) Thus, *S*(*t*) is simply the complement of the cumulative mortality function introduced above: *M*(*t*) = 1 − *S*(*t*). It is useful to introduce a probability distribution, or event density, *p*(*t*) ≡ −d*S*(*t*)/d*t*, which is the incremental fraction of firm deaths occurring in a time interval from *t* to *t* + Δ*t*. Using these definitions, we write2.10
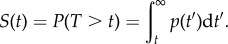
We then see that the *hazard rate*, *λ*(*t*), introduced in equation (2.1), is the normalized mortality rate at time *t*: *λ*(*t*) = −d ln*N*(*t*)/d*t*,2.11
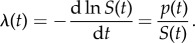
Finally, it is often useful to introduce the *cumulative* hazard function, *Λ*(*t*), defined as2.12
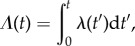
in which case *S*(*t*) = e*^−^*^*Λ*(*t*)^, the analogue of equation (2.3). Note that for there to be no survivors at very large times, i.e. *S*(*t* → *∞*) = 0, *Λ*(*t*) must become infinite and the integral in equation (2.12) consequently diverges with *t*.

The case when *λ* is constant, discussed above, is a simple example of this behaviour; it straightforwardly leads to the conventional exponential distributions, *S*(*t*) = e^−*λt*^ and *p*(*t*) = *λ*e*^−*λ*t^*, and the cumulative hazard function increasing linearly with time: *Λ*(*t*) = *λt*. These quantities can be used in practice as targets of empirical estimation, both for parametric estimation, where a form of *p*(*t*) is assumed, or non-parametric when *p*(*t*) need not be specified and fewer assumptions are necessary.

### 2.3. Maximum-likelihood estimator for constant hazard rate

To show how survival analysis can be used to generate an improved estimate of an assumed constant *λ* by including right-censored data, consider the construction of a maximum-likelihood estimator (MLE; e.g. [5]). For each firm, *i*, consider the time interval *t_i_* ≤ *T*, during which the firm is observed. The likelihood of the company disappearing (dying) within the observation window is given by *L_i_* = *p*(*t_i_*) = *λ*(*t_i_*)*S*(*t_i_*), whereas the likelihood that its lifespan exceeds *t_i_* is *L_i_* = *S*(*t_i_*). These expectations can be combined into a single formula2.13
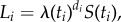
where *d_i_* = 0 if the firm is still alive at the end of the period *t_i_* = *T*, or *d_i_* = 1 if the company disappears within the observation window. The total likelihood over all firms, each taken to be a statistically independent event, is the product2.14
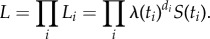
This equation is equivalent to2.15
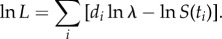
Assuming a constant hazard rate, *λ*, independent of *t_i_*, we can derive its MLE by demanding d ln *L*/d*λ* = 0. Since *S*(*t_i_*) = e*^−*λ*t_i_^*, this gives the MLE for *λ*2.16
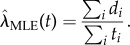
Note that 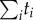 is the total number of years lived by all firms within the observation interval, *T*, and that the variance in this estimator is given by 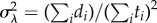. This leads to the estimate *λ* = 0.058 yr*^−^*^1^, obtained still under the assumption of constant hazard rate but now including right-censored firms. The 95% confidence interval is 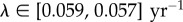. For the constrained dataset, our estimate is *λ* = 0.051 yr*^−^*^1^ with 95% confidence interval 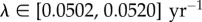.

### 2.4. Kaplan–Meier and Nelson–Aalen non-parametric estimators

We now explore non-parametric estimators, first introduced by Kaplan & Meier [38]. The advantage of these estimators is that they allow us to forego the assumption of a constant hazard rate.

Let *D*(*t_i_*) denote the number of firms dying at time *t_i_* (within the 1 year time resolution of the observed data) and *N*(*t_i_*) the number of firms still alive at that time. The number of survivors is therefore *N*(*t_i_*) − *D*(*t_i_*) so an estimate of the probability of surviving the *i*th hazard is given by [*N*(*t_i_*) − *D*(*t_i_*)]/*N*(*t_i_*) which is effectively the survival function for the *i*th time window. Assuming that each of these hazards is statistically independent leads to the Kaplan–Meier estimator for the survival function, *S*(*t*)2.17
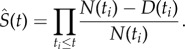
This expression can be shown to be the maximum-likelihood non-parametric estimator for *S*(*t*) [5]. Below, we give a more general derivation which relates it to the Nelson–Aalen estimator of the hazard rate.

**Figure 4.**
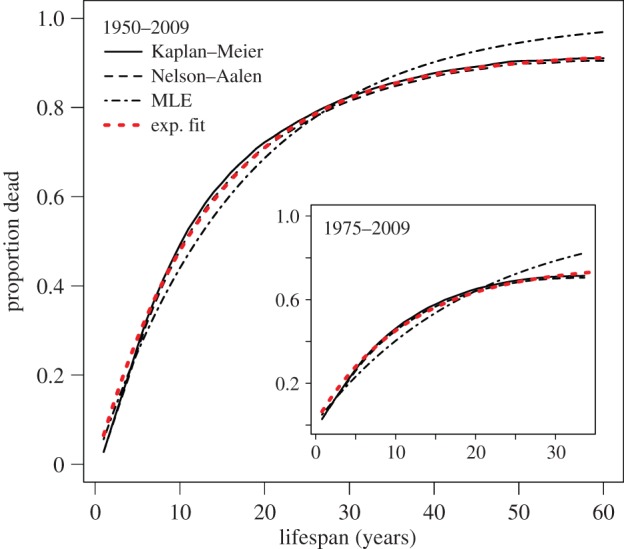
Non-parametric estimators of firm mortality versus lifespan. The mortality function *M*(*t*) = 1 − *S*(*t*) obtained from both the Kaplan–Meier and Nelson–Aalen non-parametric estimators for the full and restricted (inset) datasets is well fit by an exponential curve with constant hazard rate. Note however that the curves obtained via both non-parametric estimators deviate from the maximum-likelihood estimate for *λ*, especially for long lifespans.

We use equation (2.17) to estimate the mortality function, *M*(*t*) = 1 – *S*(*t*), as shown in figure 4 and electronic supplementary material, figure S2. Though the resulting curves are non-parametric, an exponential with constant *λ* fits the data well for the full and constrained datasets.

The use of discrete (yearly), rather than continuous, data may contribute a small upward bias, as we are effectively assuming that firm death occurs at the end of the year. It should be noted that the fitted exponential curve includes a constant equal to the proportion of firms that remain alive at the end of the observation period, limiting the potential for estimating firm immortality. In fact, the importance of its inclusion suggests that more firms may have extremely long lifespans than an assumption of constant hazard would suggest.

The Nelson–Aalen estimator [39,40] focuses on a non-parametric estimate for the cumulative hazard, *Λ*(*t*), rather than the survival function, *S*(*t*). In discrete form, the hazard rate, *λ*(*t*) ≡ −d ln*N*(*t*)/d*t*, becomes *λ*(*t_i_*) = *D*(*t_i_*)/*N*(*t_i_*)Δ*t_i_* so the cumulative hazard, equation (2.12), becomes *Λ*(*t*) = ∑*_i_λ*(*t_i_*)Δ*t_i_*, which forms the basis of the Nelson–Aalen cumulative hazard estimator2.18
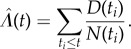


The corresponding estimator for the survival function, *S*(*t*) = e*^−^*^*Λ*(*t*)^, can therefore be expressed as2.19
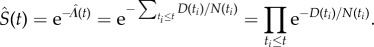


In the limit 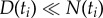 the exponential, 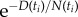, is well approximated by [1 − *D*(*t_i_*)/*N*(*t_i_*)] = [*N*(*t_i_*) − *D*(*t_i_*)]/*N*(*t_i_*), in which case, equation (2.19) reduces to the Kaplan–Meier expression, equation (2.17). Corrections to (2.17) arise primarily from hazards occurring at late times *t_i_* close to *t* when the number dying becomes comparable to the number still alive. Thus, estimates for *λ* obtained from the Kaplan–Meier estimator are not expected to differ significantly from those generated using the Nelson–Aalen approach.

The cumulative rate, estimated from equation (2.17), as a function of company age is shown in electronic supplementary material, figure S3. Figure 4 and electronic supplementary material, figure S4, show the corresponding mortality curves, obtained via *M*(*t*) = 1 − *S*(*t*), obtained for each estimator. Exponential curves fitted to the Kaplan–Meier and Nelson–Aalen mortality curves offer similar results, summarized in table 2.

**Table 2.** Exponential fits to mortality data by estimator.

method	*M*(*t*)	95% CI
Kaplan–Meier	0.925 (1−e*^−^*^0.074*t*^)	[0.073, 0.076]
1975–2009	0.765 (1−e*^−^*^0.090*T*^)	[0.085, 0.095]
Nelson–Aalen	0.921 (1−e*^−^*^0.072*t*^)	[0.071, 0.074]
1975–2009	0.759 (1−e*^−^*^0.088*T*^)	[0.083, 0.093]

### 2.5. Half-life comparisons

As discussed above, extended windows applied to a modified exponential fit lead to a half-life estimate of approximately 7.02 years; including censored observations leads to higher estimates. The MLE 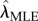, equation (2.15), gives a significantly higher half-life estimate of almost 12 years, while an exponential curve fitted to the Kaplan–Meier estimator suggests a slightly lower half-life estimate of around 10.5 years and, as predicted, the Nelson–Aalen estimator results in a similar estimate of 10.8 years. A comparison of these various half-life values as well as 95% confidence intervals is given in table 3.

**Table 3.** Half-life estimates by estimator.

method	estimate	95% CI
frequency windows	7.02	[6.34, 7.73]
maximum likelihood	11.94	[11.93, 11.95]
Kaplan–Meier	10.46	[10.23, 10.70]
Nelson–Aalen	10.83	[10.60, 11.07]

Within 95% confidence intervals the Nelson–Aalen and Kaplan–Meier estimates of *λ* are not significantly different, as expected. While the MLE estimate is lower in magnitude, only the estimate obtained without censored observations is different in a statistically significant sense. When we constrain the window of observation, estimates of *λ* obtained from an exponential curve fitted to the Kaplan–Meier and the Nelson–Aalen curves are significantly different, but the magnitudes are not widely divergent. The half-life estimates obtained from the constrained window also remain within 2 years of the estimates obtained from the full dataset.

## 3. Discussion

Our results support the hypothesis that mortality rates for publicly traded companies are approximately age independent. The current analysis provides one of the largest and most comprehensive studies of this kind, both in terms of numbers of firms and timespan covered. Our main result is the estimation of an approximately constant hazard rate for the death of publicly traded firms, especially as the window of observation reaches two decades or longer. We also find that the distribution of lifespans is consistent across methods of estimation, including those that assume a parametric form for the survival function of firms and those that do not. While our measure of half-life varies with the method used to incorporate censored observations, a half-life of approximately 10 years is typical for the firms in our dataset.

As noted in Material and methods and summarized in table 1, the ‘death’ of firms is most often associated with mergers and acquisitions rather than with an event that leads to its organizational demise, as in biological organisms. We have deconstructed our data into these two broad categories of ‘death’ and find that both follow an approximately constant hazard rate with slightly different values of their mortality.

When companies disappear through mergers and acquisitions, they often persist in some form as part of other organizations. This is an important point because it associates death events with organizational splits and mergers that may make sense in the light of the structure of transaction costs, rather than the failure to be productive and disappear. Furthermore, a constant hazard rate, independent of age, suggests that at each stage of a firm's life cycle there is a similar probability to being acquired. This is consistent with a picture of mergers and acquisitions that, at least initially, only loosely ties the two firms involved together. In this way, if the boundaries of the *ex-ante* firms were set by the balance of their internal to market transaction costs, the new merged corporation will be approximately neutral in terms of net gains.

This relatively frequent and approximately neutral fusion–fission dynamics of firms suggests that their evolution and growth is dictated to a large extent not only by their internal growth potential but also by the economies and efficiencies that merging firms may be able to obtain. Thus, the risk of being acquired remains constant in each time period of a company's lifecycle, implying that most companies will disappear within a finite time, typically of the order of a decade.

Besides these general arguments there may be other reasons for the trends observed such as lower mortality rates in the first years after a firm becomes publicly traded. Bruderl & Schussler [16] suggest that liability of newness is more likely a product of selection than of adaptation. If this is true, then counting incorporation as birth may reflect a selection bias for more successful firms. Moreover, their initial public offerings supply these companies with an injection of capital that may ensure their viability for a number of years. This result suggests that market mechanisms allow successful companies to buffer against extrinsic age-dependent sources of mortality by either raising capital or acquiring skill-sets of competitors.

Our results potentially conflict with the well-known fact that several companies, especially in Japan and Europe, have lived for hundreds of years (http://en.wikipedia.org/wiki/List_of_oldest_companies). Cohort data are almost certainly unavailable in such cases, but extrapolating our result that the hazard rate is approximately constant, *λ* ∼ 0.1 yr*^−^*^1^, predicts that the probability of surviving 100 years is approximately 4.5 × 10*^−^*^5^ and for 200 it is approximately 10*^−^*^9^. So, for example, if there are 10^8^ companies in the world and they obey the same dynamics as the cohorts we analysed, then 4500 might be expected to survive 100 years, but *none* for 200. The analysis of datasets with longer timespans may shed light on the dynamics of long-lived firms and on the ultimate question of potential firm ‘immortality’. In this regard, it is poignant to note that the purported longest lived company in the world, Kongo Gumi, founded in AD 578, went into liquidation in 2006 when its assets were purchased by the Takamatsu Corporation.

An interesting set of questions deals with the potential variation of lifespan statistics by sector of activity. We performed these analyses and continue to see evidence of an exponential distribution of lifespans when we break down our data by economic sector and also when we fine-grain our data and look at each cohort by year of birth. Size at birth, however, does have a clear positive correlation with lifespan (electronic supplementary material, figure S6). Further analyses that continue this line of investigation may offer insight as to the mechanisms behind the result of (approximately) constant firm death hazard and its consequences for theories of the firm.

## 4. Material and methods

### 4.1. Datasets

Data on publicly traded companies were obtained from the *Compustat North America* and *Compustat Historical* databases, compiled by Standard & Poor's [36]. Our datasets cover the period of 1950–2009 and contain most financial information for North American and overseas American Depositary Receipt firms reported in their income statements and balance sheets, filed to the US Securities and Exchange Commission. A total of 28 853 publicly traded companies are included in the database. From these, we excluded 2292 that did not report any sales over the 60-year timespan. We also noted that 6868 companies were listed (alive) either in 1950 or in 2009, with 160 of those companies reporting sales for the full 60-year span of the dataset. These can be considered ‘censored’ in that we know the *minimum* number of years such firms must have lived, but we do not know their full lifespans. When we implement survival analysis to include censored companies, we obtain a sample size of 26 561 companies.

### 4.2. Definitions

Our analysis of firm mortality and longevity differs from prior research in the definition of firm *birth* [5]. We define ‘birth’ to occur not at a company's founding, but rather when it first reports sales in the *Compustat* database. We take ‘death’ to occur in the year when a company stops reporting sales. This definition is similar to the Bureau of Labor Statistics Business Employment Dynamics measures of entries, which include mergers, takeovers and industrial reclassification [41]. This definition of death leads us to include very different types of events in a single category (table 1). As noted by Carroll & Delacroix [15], this broad definition of death will affect the conclusions we can draw from our data, as an instance of firm death does not necessarily connote failure. For each company, we define *lifespan* to be the total number of years for which the company reports non-zero sales. Sadeghi [41] reports the difficulties of basing such measures on a company's reported employment, and we find a number of instances of companies reporting sales data but not employment. We expect that sales reports will be a more accurate measure of the company's existence in that year. There are a number of companies that fail to report for several years between years of activity. Such cases of re-entry are not counted as additional new births or deaths; the additional years are simply added to the total lifespan. This is in keeping with methods used by several other researchers [5]. As a metric of mortality that is closely related to lifespan, we use the term *half-life*, defined as the time it takes for half of the firms in a given cohort to die (following the above definition of death). For survival analysis, this half-life corresponds to the age *t* by which the cumulative mortality fraction *M*(*t*) = 0.5 (50%).

### 4.3. Survival biases and subsampling

While the later decades of the *Compustat* database are generally considered to be free of survival bias, Ball & Watts [42] note that the historical data do have problems of survival bias. In figure 1, we observe that almost no firms die in the first 20 years of the dataset. Of those firms already alive in 1950, even the shortest lived firm has a lifespan of at least 19 years. To account for the effects of this bias, we ran our analysis both on the entire dataset and on a set limited to firms reporting sales between 1975 and 2009. This reduces our dataset to the entire lifespans of 14 268 firms and the censored lifespans of 11 626 firms. A comparison between analysis of the entire sample and this reduced set suggests that the effect of survival bias is limited. This is likely because the first 20 years comprise a very small proportion of the entire dataset.

## Supplementary Material

Supplementary online material
