# Supplementary material for: The mortality of companies
Source: J R Soc Interface. 2015 May 6;12(106):20150120. doi: 10.1098/rsif.2015.0120 (PMC4424689; doi:10.1098/rsif.2015.0120)
Supplement: Supplementary online material [file rsif20150120supp1.pdf]

The Mortality of Companies

Supplementary Online Material

M. G. Daepp[1,2], M. J. Hamilton[1,3], G. B. West[1], L. M. A. Bettencourt[1]

[1]Santa Fe Institute, Santa Fe, New Mexico, USA.
[2]Integrated Studies in Land & Food Systems, University of British Columbia, Vancouver, BC, CA.
[3]School of Human Evolution and Social Change, Arizona State University, Tempe, AZ, USA.
E-mail: marcusj@santafe.edu

January 20, 2015

Contents:

Supplementary Figures S1, S2, S3, S4, S5.

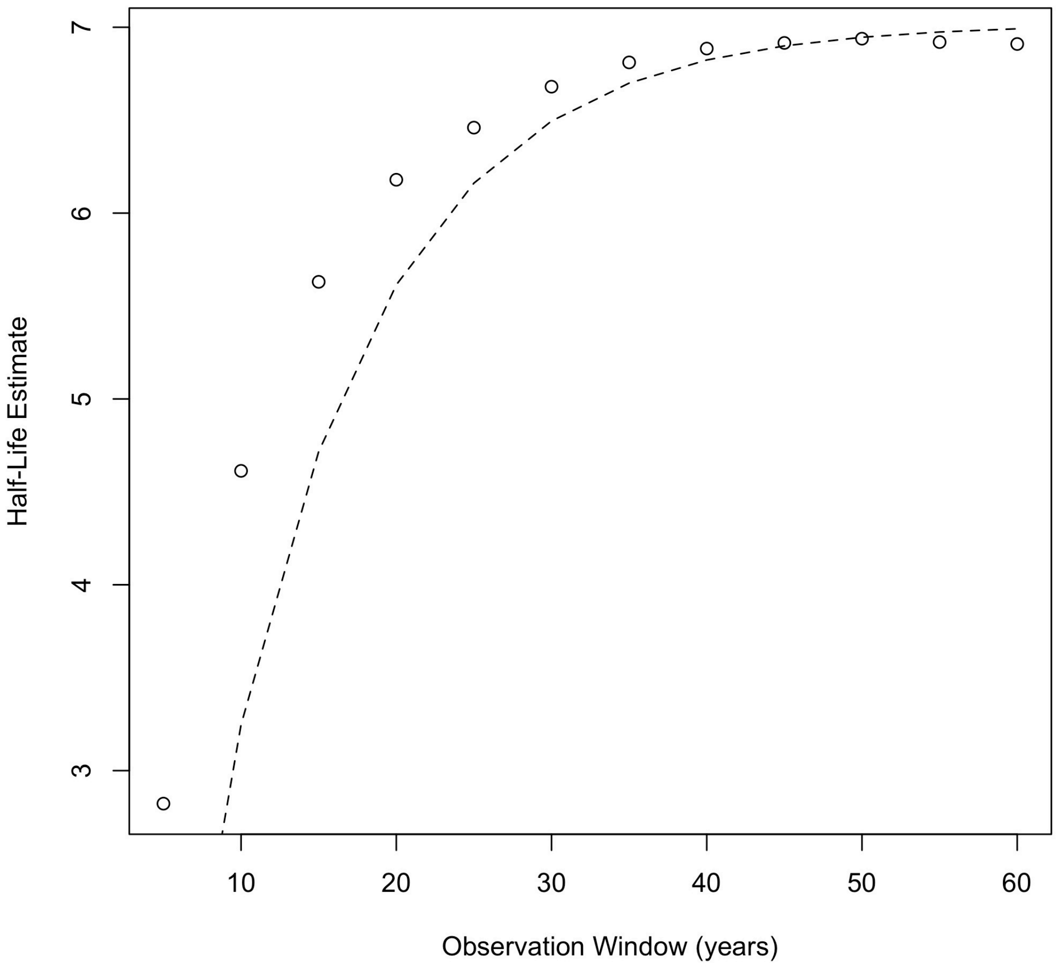

Figure S1: **Predicted Half-Life Estimates from Constant Hazard Model**. The curves of Figure 2 result in estimates for the constant hazard rate (over its time interval) λ. These are then entered into the cumulative distribution function to determine the half-life of the set of firms with lifespans of T or less. The resulting curve is well fit is $t_{1/2} = \frac{\ln 2}{\lambda}[1 - \frac{\ln(1+\exp(-\lambda T))}{\ln(2)}]$, with $\lambda \approx 0.0988$ and 95% confidence intervals [0.0896, 0.1094]. This predicts that $T \to \infty$, it will take approximately $t_{1/2} = 7.019$ years for half of all firms to die.

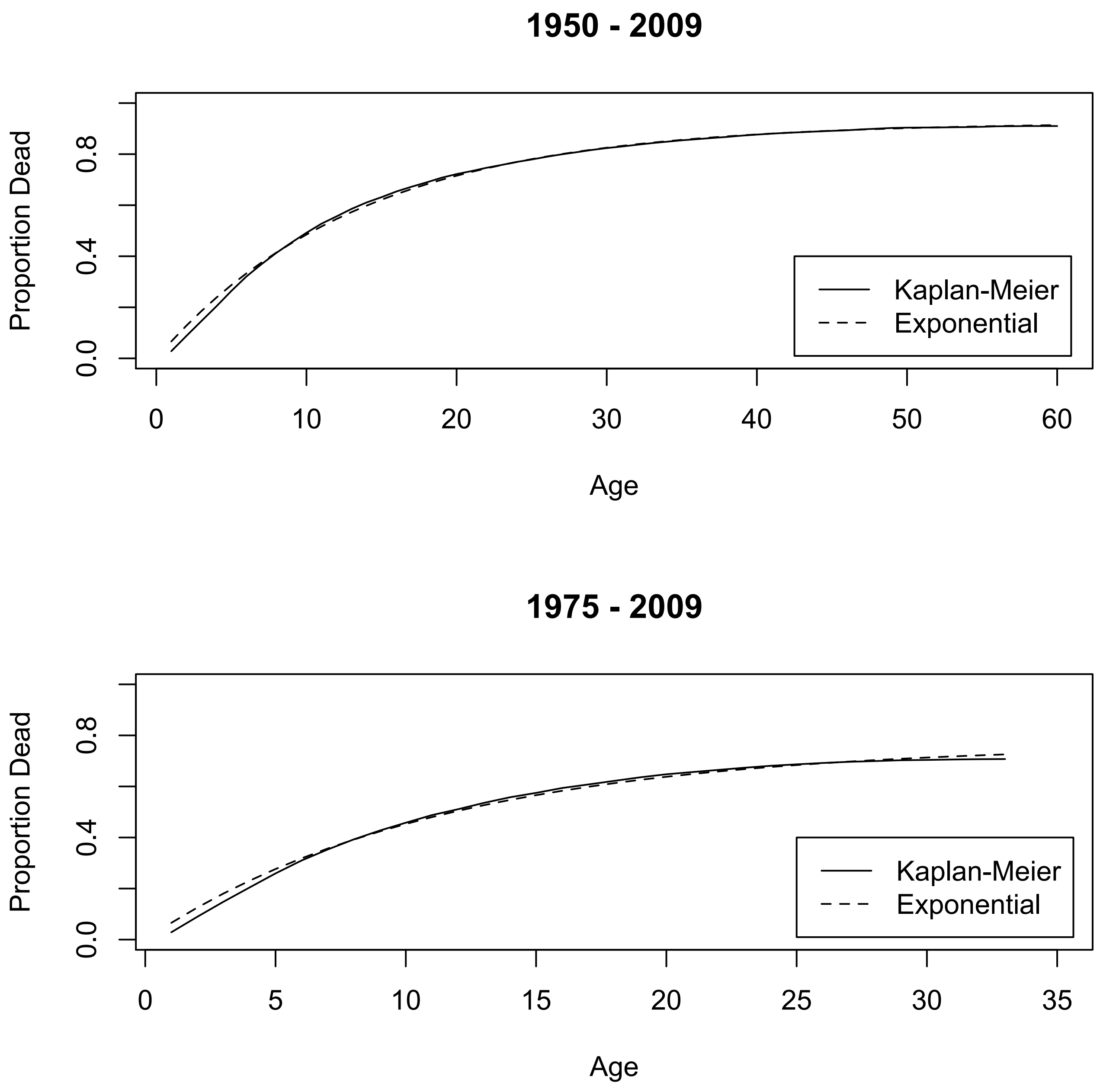

Figure S2: **The Kaplan-Meier Mortality Curve**. The nonparametric Kaplan-Meier estimator results in a form of the mortality curve, $M(T) = 1 - S(T)$. The curves fitted to these distributions are $M(T) = 0.925(1 - e^{-0.074T})$ with 95% confidence intervals [0.073, 0.076] and [0.918, 0.931]. For the constrained version, we estimate $M(T) = 0.765\left(1 - e^{-0.090T}\right)$, with 95% confidence intervals [0.749, 0.782] and [0.085, 0.095].

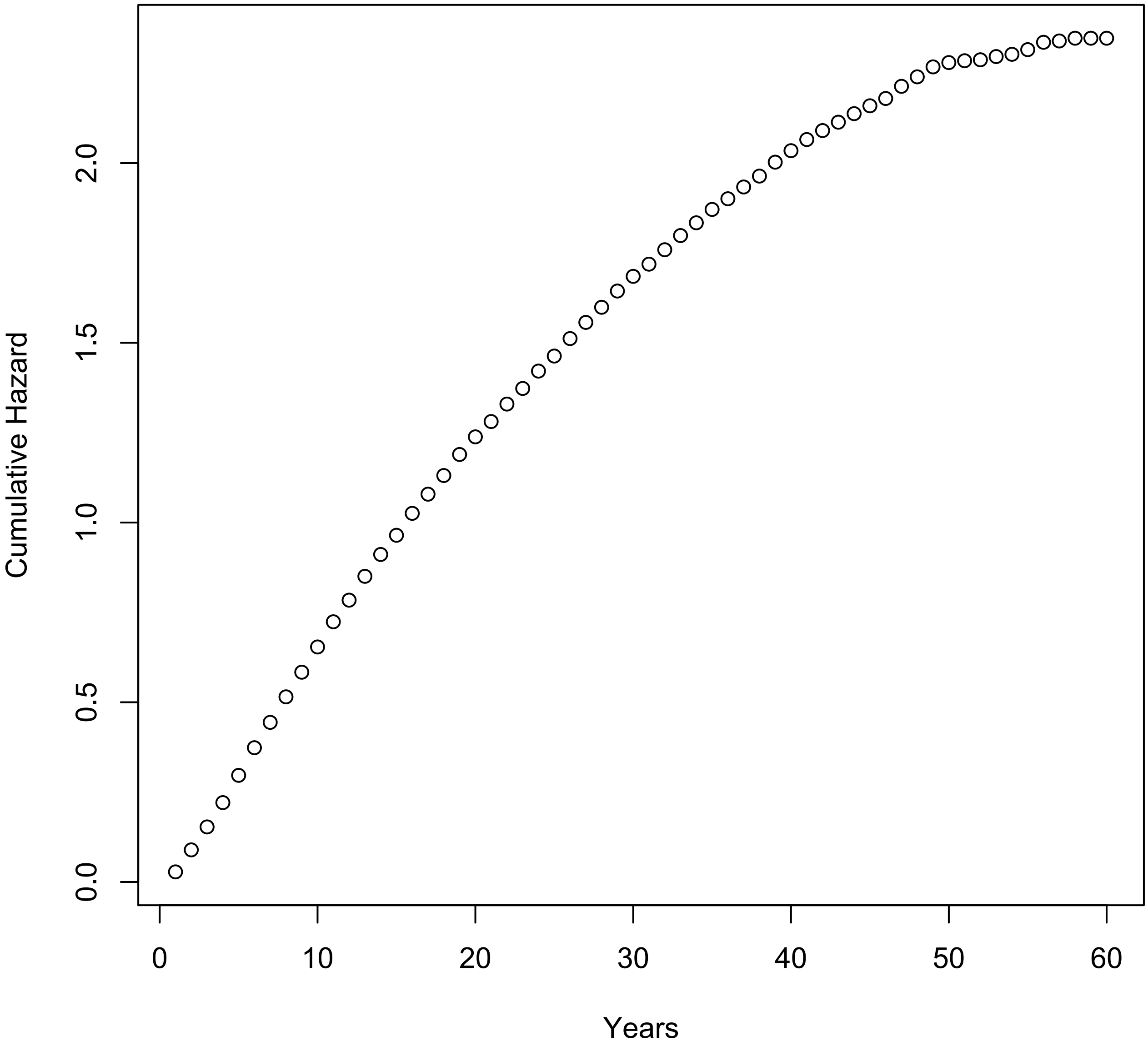

Figure S3: **The cumulative death hazard Λ computed via the Nelson-Aalen estimator.** A linear cumulative hazard Λ in time is a sign of constant λ. The slight concavity of the cumulative hazard therefore points to a decreasing λ and increasing longevity with age.

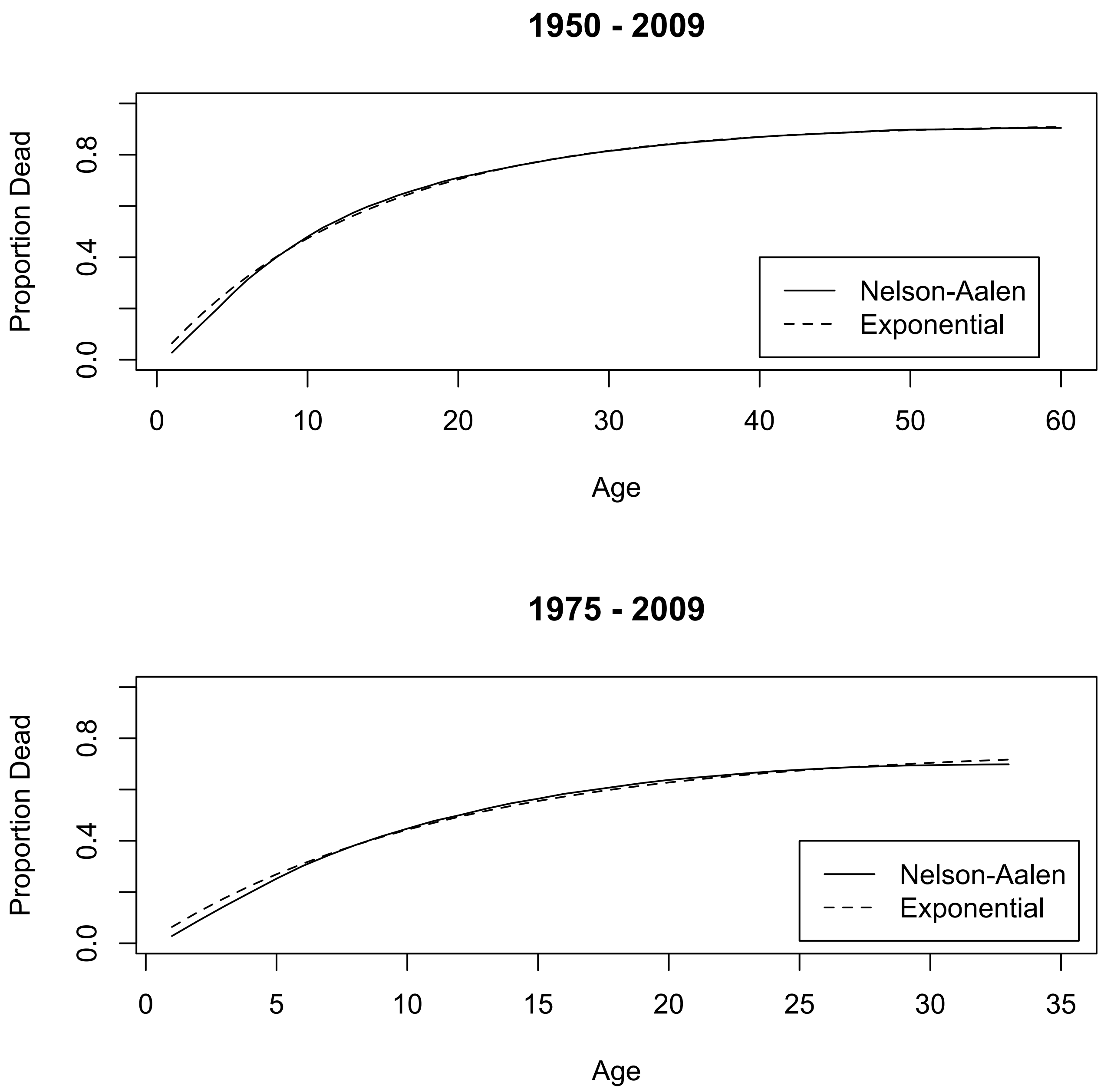

Figure S4: **The Nelson-Aalen Mortality Curve** We constructed the mortality curve from the cumulative hazard, via $M(T) = 1 - S(T)$, with $S(T) = e^{-\Lambda(T)}$. For the cumulative distribution, $M(T) = 0.92\left(1 - e^{-0.072T}\right)$ with a 95% confidence intervals [0.915, 0.927] and [0.071, 0.074]. The constrained equation has the estimated fit $M(T) = 0.759\left(1 - e^{-0.088T}\right)$ and 95% confidence intervals [0.743, 0.776] and [0.083, 0.093].

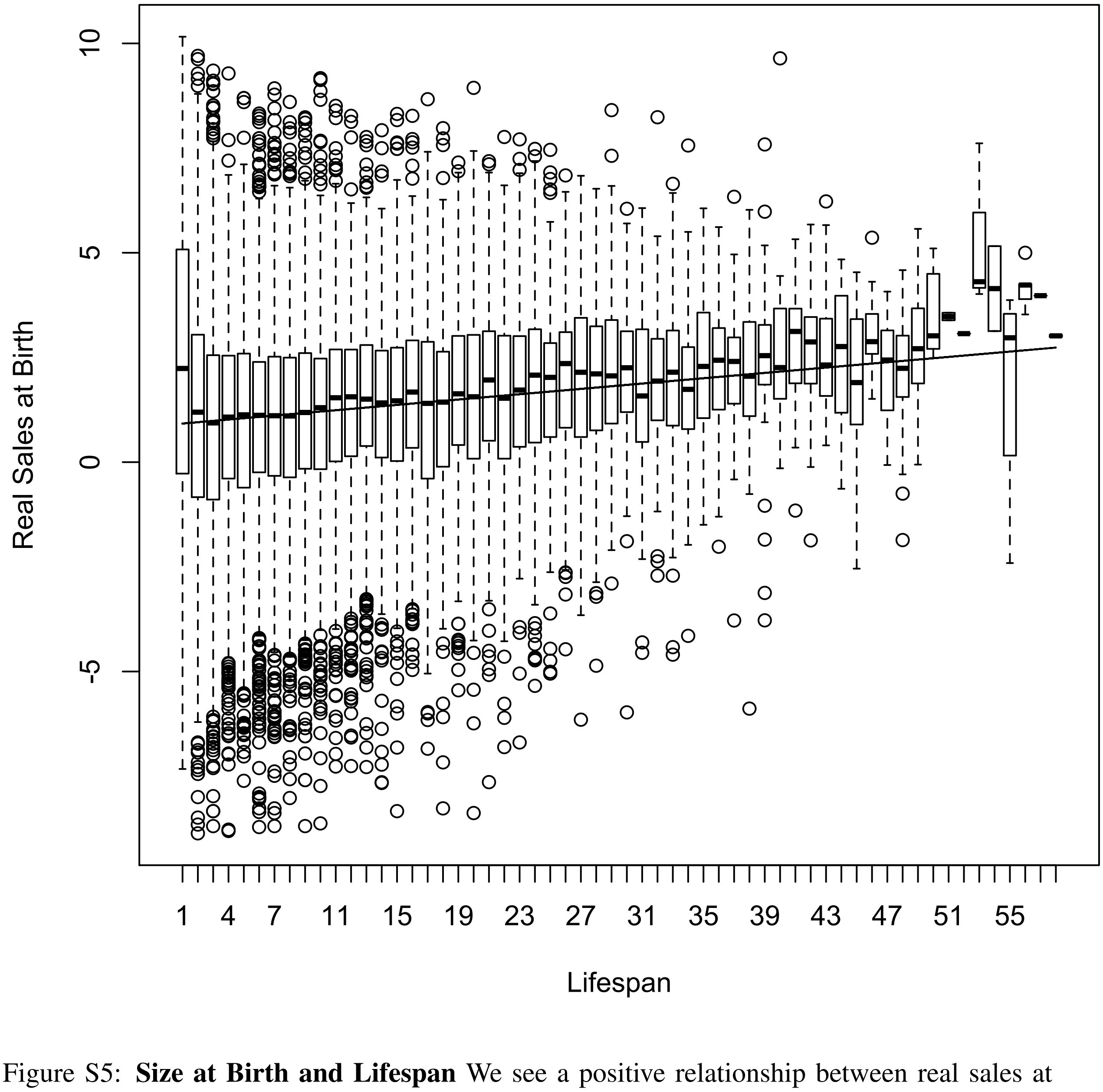

Figure S5: **Size at Birth and Lifespan** We see a positive relationship between real sales at birth and the lifespan of all companies that were born and died within our sixty-year window. A fit of $log(BirthSales) = 0.89 + 0.0318(Lifespan)$ with a 95% confidence intervals [0.8338, 0.9462] and [0.0277, 0.0359].
